# Development of monoclonal antibodies and serological assays specific for *Barley yellow dwarf virus* GAV strain

**DOI:** 10.1186/s12985-015-0367-4

**Published:** 2015-09-04

**Authors:** Na Li, Zhe Chen, Yan Liu, Yong Liu, Xueping Zhou, Jianxiang Wu

**Affiliations:** State Key Laboratory of Rice Biology, Institute of Biotechnology, Zhejiang University, Hangzhou, Zhejiang 310058 China; Institute of Plant Protection, Chinese Academy of Agricultural Sciences, Beijing, 100081 China; Hunan Plant Protection Institute, Changsha, 410125 China

## Abstract

**Background:**

*Barley yellow dwarf virus* (BYDV) is one of the most devastating plant viruses and belongs to a ubiquitous plant virus group. In China, four BYDV strains (GPV, GAV, PAV and RMV) have been identified based on their specific aphid vectors and serological properties. Among the four identified strains, the GAV is the most common BYDV strain in China. To diagnose, forecast of BYDV GAV, two reliable serological assays for BYDV GAV detection were established.

**Methods:**

We purified virion from a confirmed BYDV GAV source and used it as the immunogen to produce monoclonal antibodies against the virus. Using the hybridoma technology, three highly specific murine monoclonal antibodies were produced and two serological assays [antigen-coated-plate enzyme-linked immunosorbent assay (ACP-ELISA) and dot enzyme-linked immunosorbent assay (dot-ELISA)] were established for the BYDV GAV detection.

**Results:**

All three monoclonal antibodies reacted strongly and specifically with the BYDV GAV strain in crude leaf extracts. Titers of the monoclonal antibodies in ascitic fluids were up to 10^−7^ by indirect-ELISA. These three monoclonal antibodies (18A1, 18A9 and 12A11) all belonged to the isotype IgG1, kappa light chain. The highest dilution points for the three antibodies during the ACP-ELISA using infected crude leaf extracts were 1:163,840, 1:81,920 and 1:81,920 (w/v, g · mL^−1^), respectively. Result of dot-ELISA showed a successful detection of BYDV GAV strain in 1:5,120 (w/v, g · mL^−1^) diluted wheat leaf crude extracts. Analysis of 22 field wheat leaf samples and 33 aphid samples from the Shaanxi Province in China, using the two newly developed assays confirmed the presence of BYDV GAV in about 80 % of the wheat samples and 18 % of the aphid samples.

**Conclusions:**

All three monoclonal antibodies are highly sensitive and specific to the BYDV GAV. The two newly developed serological assays are simple and effective. These two assays, particularly the dot-ELISA, are useful for high throughput detection of BYDV GAV in host plants and aphid vectors.

## Background

*Barley yellow dwarf virus* (BYDV) causes substantial losses in wheat (*Triticum aestivum* L.), barley (*Hordeum vulgare* L.), and oat (*Avena sativa* L.) production, and occasionally in rice (*Oryza sativa* L.) and maize (*Zea mays* L.) production [1]. BYDV is considered as one of the most devastating plant viruses and belongs to a ubiquitous plant virus group [2]. BYDV-caused barley yellow dwarf disease was first reported as a disease transmitted by aphid vectors in 1951 [3]. BYDV is known as a type member in the *Luteovirus* group, family *Luteoviridae* [4]. It is a phloem-limited virus transmitted by several cereal aphid species in a circulative-nonpropogative, persistent manner [2]. BYDV strains showed high degrees of aphid vector specificities and a single BYDV strain can only be transmitted efficiently by one or a few aphid species [5]. Earlier studies by Rochow determined the presence of five BYDV strains (RPV, MAV, RMV, SGV and PAV) in the US [5, 6]. In China, four strains (GPV, GAV, PAV and RMV) of BYDV have been reported based of the specificities of their aphid vectors and serological properties [7]. The Chinese GAV and PAV strains showed strong serological cross-reactions with the MAV and PAV strains from the US, and the Chinese RMV strain is similar to the American RMV strain [7]. The GPV strain reported in China is transmitted by *Schizaphis gramium* and *Rhopalosiphumpadi* and is not serologically related to the five American BYDV strains [8].

BYDV is known to infect multiple grasses and cereal crops like barley and wheat, and are often referred to as barley yellow dwarf disease and wheat yellow dwarf disease [4]. Symptoms on the BYDV-infected plants can be similar to that caused by nutrient or water deficiency. In addition, symptoms on the BYDV-infected wheat vary significantly among different wheat cultivars, age of the plant when it becomes infected, virus strains, aphid vectors as well as environmental conditions. In general, the yellowing disease phenotypes started from the tips of leaves and extended downward to whole leaf leading to severe stunting of the plant. The infected plants also showed reduced number of ears and grains [4, 9, 10]. BYDV was first reported in the Shaanxi Province of China in 1960. In recent years, BYDV GAV-caused wheat yellow dwarf disease has observed throughout the northern and north-western regions of China. It was reported that outbreak of BYDV in field often coincided with a high population of viruliferous aphid vectors on the overwintered host plants during the most susceptible stage of the wheat crops and the changes in cultivation practices [11]. BYDV was shown to accumulate in phloem cells and BYDV viroplasm, virion aggregates and BYDV specific tubular structures were observed in both infected plant and aphid cells [12].

Genome of BYDV is approximately 5700 nucleotides in length and contains six open reading frames (ORFs) [13]. Genome organization of BYDV GAV is similar to that of BYDV MAV [8]. ORF1 of BYDV GAV encodes a 39 kDa protein with an unknown function and is translated from the first AUG codon in the genome. ORF2 encodes the RNA-dependent RNA polymerase (RdRp) of the virus. ORF3 in the subgenomic RNA encodes the viral coat protein of about 22 kDa. ORF4 is entirely within the ORF3 and encodes a protein of 17 kDa that is necessary for BYDV movement between host cells. ORF5 encodes a 50 kDa protein and is translated by in-frame readthrough of the coat protein stop codon. It was reported that the 50 kDa protein fused to the CP to form a 60 kDa readthrough protein. However, this reported size is clearly smaller than the predicted 72 kDa readthrough protein [14]. The protein encoded by ORF6 of BYDV GAV is about 4.3 kDa with an unknown function [15]. We had searched the genomic information about GAV, PAV, GPV and RMV strains in GeneBank and obtained four genomic sequences (NC004666 for GAV, FM865413 for GPV, AY855920 for PAV and NC021484 for RMV). The nucleotide sequence identity in the whole genome level for those four BYDV strains was low, just 34.1–75.2 %. The amino acid sequence identity of the 72 kDa readthrough protein in those four BYDV strains was only 7.7–62.6 %. So, the readthrough protein is most appropriate to produce monoclonal antibodies specific for BYDV GAV.

Procedures currently used to detect BYDV infection include reverse transcription polymerase chain reaction assay (RT-PCR) [16], dot blot nucleic acid hybridization [17], enzyme-linked immunosorbent assay (ELISA) and immunoprinting using specific polyclonal antibodies [16, 18, 19]. Among these procedures, the serological assays are the more suitable assays for a high throughput test of field samples. Because a successful serological assay depends largely on the availability and specificity of the antibody, we decided to produce highly sensitive and specific murine monoclonal antibodies against BYDV GAV using the hybridoma technology. We have also developed an antigen-coated enzyme-linked immunosorbent assay (ACP-ELISA) and a dot enzyme-linked immunosorbent assay (dot-ELISA) for the detection of BYDV GAV using these monoclonal antibodies. The evidence provided in this paper showed the usefulness of these two assays for field-collected wheat and aphid samples. We believe that these serological assays, particularly the dot-ELISA, can be used for high throughput detection of BYDV GAV infection during field surveys at a low cost.

## Results

### Virus purification

**Fig. 1:**
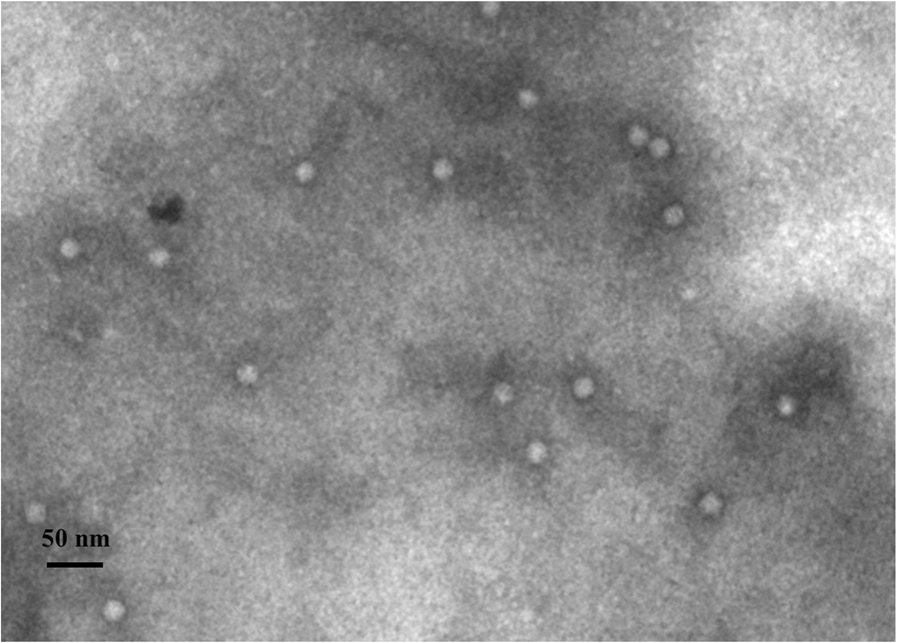
Electron micrograph of purified *Barley yellow dwarf virus* (BYDV) GAV particles. Virus particles were purified from the BYDV GAV-infected wheat plant tissues through differential centrifugation. Purified virus samples were loaded on formvar-coated grids, negatively stained with 2 % (w/v, g · mL^−1^) phosphotungstic acid and examined under an electron microscope. Bar = 50 nm

Virion of BYDV GAV was purified from infected wheat leaf tissues harvested at 30 days post virus inoculation (dpi) by differential centrifugation. Icosahedral virion of approximately 30 nm in diameter was observed in the purified virus preparations under a transmission electron microscope (Fig. 1).

### Preparation and characterization of monoclonal antibodies specific for BYDV GAV

Purified BYDV GAV virion was used to immunize BALB/c mice (*mus musculus*). After the forth immunization, spleen cells of the immunized mice were obtained and used for hybridoma production. Three hybridoma lines (18A1, 18A9 and 12A11) secreting monoclonal antibodies against BYDV GAV were obtained and then injected intraperitoneally to BALB/c mice to produce ascitic fluids. The IgG yields of the ascitic fluids containing these antibodies were 10.23, 9.8 and 7.65 mg · mL^−1^, respectively (Table 1). The immunoglobulin classes and subclasses of the three monoclonal antibodies were isotyped as IgG1, kappa light chain (Table 1). The titers of the three monoclonal antibodies in ascitic fluids were up to 10^−7^ by an indirect-ELISA.

**Table 1:** Characterization of BYDV GAV monoclonal antibodies

MAbs	Isotype	Ascites titer	IgG yield (mg · mL^−1^)
18A1	IgG1, κ chain	10^-7 a^	10.23
18A9	IgG1, κ chain	10^−7^	9.8
12A11	IgG1, κ chain	10^−7^	7.65

^a^The monoclonal antibody titer was the highest dilution that yielded an absorption value above 0.3 at 30 min after the addition of the substrate at room temperature

**Fig. 2:**
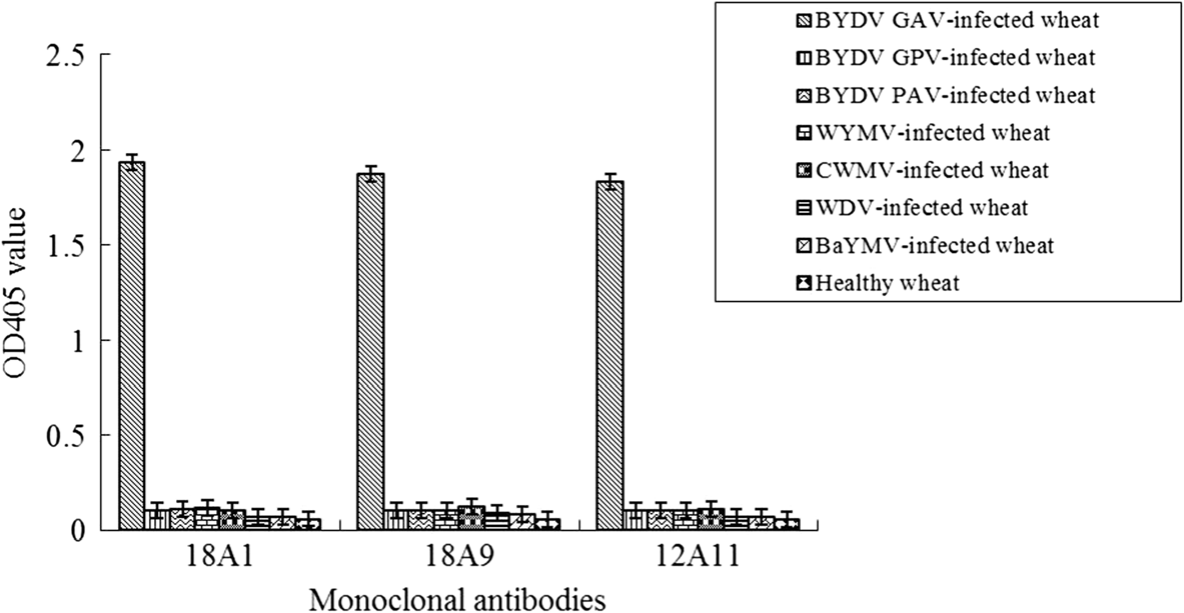
Determination of BYDV GAV monoclonal antibody specificities through ACP-ELISA. BYDV GAV-, BYDV GPV-, BYDV PAV-, WYMV-, CWMV-, WDV- or BaYMV-infected wheat plant extracts (BYDV GAV, BYDV GPV, BYDV PAV, WYMV, CWMV, WDV and BaYMV) were used in this assay. Crude extract from a healthy wheat plant was used as a negative control for the assay. Sample arrangements for antibodies 18A9 and 12A11 are the same as that for antibody 18A1. Dilutions of the three antibodies were 1:5,000, 1:6,000 and 1:5,000 (v/v), respectively

**Fig. 3:**
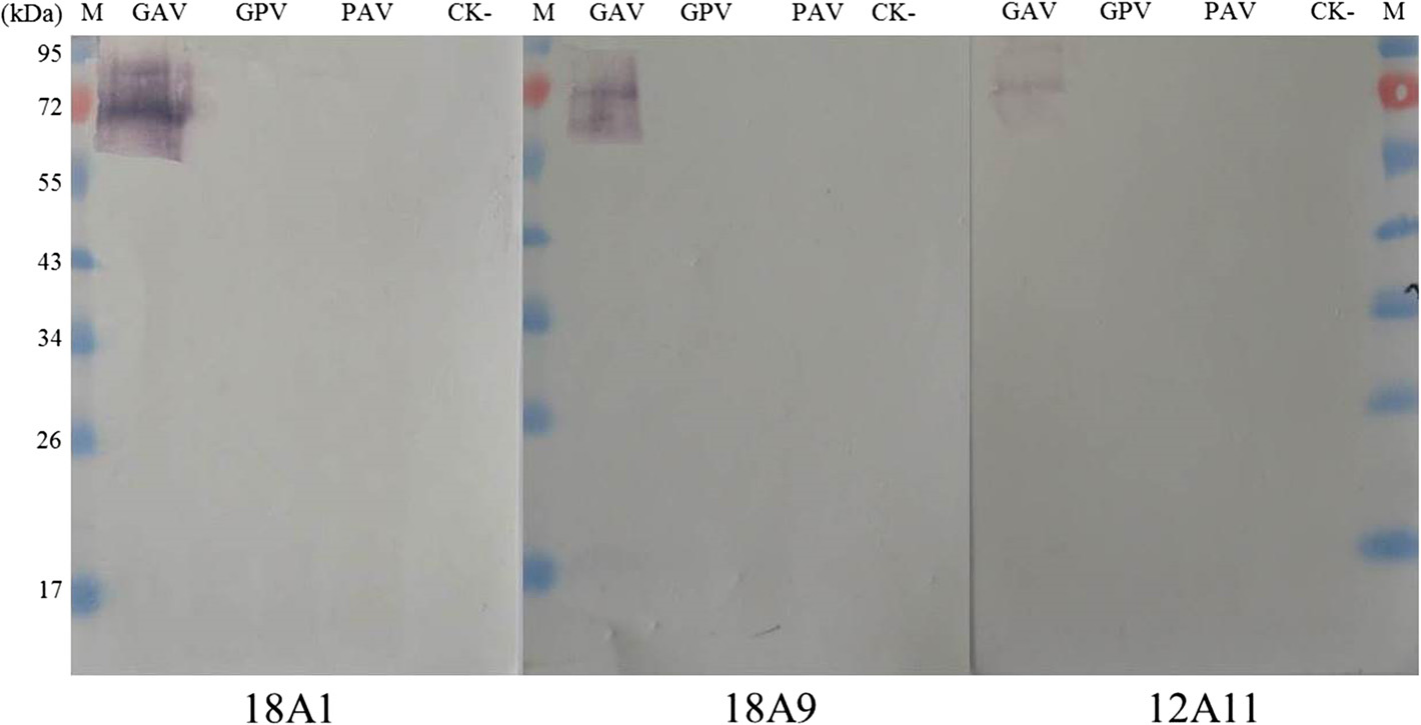
Western blot analyses of BYDV GAV infection using monoclonal antibodies. BYDV GAV-, BYDV GPV- or BYDV PAV-infected wheat leaf extracts (GAV, GPV and PAV) were used in this assay. Wheat leaf extract from a healthy plant (CK-) was used as a negative control. Dilutions of the three monoclonal antibodies were 1:5,000. Goat anti-mouse IgG/AP conjugate was used as the second antibody for the assay. M, a protein marker. The size of the protein bands are indicated on the right side of the panel

Results of ACP-ELISA showed that all the three antibodies reacted strongly with the crude extracts prepared from the BYDV GAV-infected wheat plant tissues, but had a negative reaction with the extracts from the BYDV GPV-, BYDV PAV-, *Wheat dwarf virus* (WDV)-, *Wheat yellow mosaic virus* (WYMV)-, *Chinese wheat mosaic virus* (CWMV)-, *Barley yellow mosaic virus* (BaYMV)-infected or healthy wheat plant tissues (Fig. 2). Western blot results also confirmed the specificity of the antibodies and showed that these three antibodies all reacted strongly with a single protein band of approximately 72 kDa from the BYDV GAV-infected wheat leaf extracts. This 72 kDa protein band was not observed in the samples prepared from the BYDV GPV-, BYDV PAV-infected or healthy wheat plant tissues (Fig. 3).

**Fig. 4:**
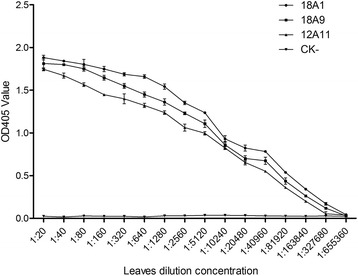
Analysis of monoclonal antibody sensitivity through ACP-ELISA. BYDV GAV-infected and healthy (CK-) wheat leaf crude extracts were two-fold diluted [1:20 to 1:655,360 (w/v, g · mL^−1^)] in a 0.05 mM sodium bicarbonate buffer and 100 μl diluted wheat extract was loaded into each well on the ELISA plate. Monoclonal antibody 18A1, 18A9 and 12A11 were diluted 1:6,000, 1:5,000 and 1:5,000 prior to the assay. Each OD_405_ value represents the mean of three independent assays at 40 min post addition of the substrate at room temperature

Sensitivities of these antibodies for BYDV GAV detection were determined through ACP-ELISA using 1:20 to 1:655,360 diluted crude extracts from the BYDV GAV-infected plant tissues. Results showed that the highest leaf extract dilutions for the three antibodies were 1:163,840, 1:81,920 and 1:81,920, respectively (Fig. 4) indicating that these antibodies were highly sensitive and specific for BYDV GAV. Consequently, monoclonal antibody 18A1 was selected for the further assays.

### ACP-ELISA for BYDV GAV detection

To establish an ACP-ELISA for BYDV GAV detection, the proper working dilutions of the monoclonal antibody 18A1 and the goat anti-mouse IgG/AP conjugate were determined using the phalanx tests. Results from three independent ACP-ELISA assays revealed that BYDV GAV could be readily detected in the greenhouse infected wheat plant tissues through this method using antibody 18A1 diluted at 1:6,000 and the goat anti-mouse IgG/AP conjugate diluted at 1:8,000. To determine the usefulness of this method for field wheat samples, crude extracts from the BYDV GAV-infected wheat plants were diluted and used in the assay. Results showed that the newly developed ACP-ELISA could be used to detect the virus in the 1:163,840 (w/v, mg · mL^−1^) diluted samples (Fig. 4). In this assay, crude extracts from the BYDV GPV-, BYDV PAV-, WDV-, WYMV-, CWMV- or BaYMV-infected or healthy wheat plants showed negative reactions (Fig. 2).

### Dot-ELISA for BYDV GAV detection

**Fig. 5:**
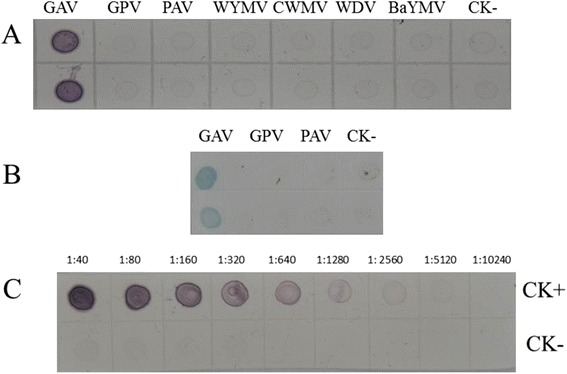
Specificity and sensitivity of the dot-ELISA. **a**, Crude extracts were prepared from the BYDV GAV-, BYDV GPV-, BYDV PAV-, WYMV-, CWMV-, WDV- or BaYMV-infected wheat plants (GAV, GPV, PAV, WYMV, CWMV, WDV and BaYMV) and blotted onto the membrane. Each dot contained 2 μl extract and each sample has two dots (up and lower). Crude extract from healthy wheat plants (CK-) was used as a negative control. **b**, Aphids fed on the BYDV GAV-, BYDV GPV- or BYDV PAV-infected wheat plants (GAV, GPV and PAV) were used for this assay. Each dot contained 2 μl extract and each sample has two dots (up and lower). Extract from aphids fed on the healthy wheat plant (CK-) was used as a negative control. **c**, BYDV GAV-infected (CK+) and healthy wheat (CK-) plants were used in this assay. Crude extracts from the GAV or CK- plants were two-fold diluted from 1:40 to 1:10,240 (w/v) in 0.01 mM PBS prior to the assay. The membrane was probed with the monoclonal antibody 18A1 followed by the goat anti-mouse IgG/AP or HRP conjugate

To establish this assay, the optimal working dilutions of the monoclonal antibody 18A1, goat anti-mouse IgG/AP or goat anti-mouse IgG/HRP conjugates were also determined by the phalanx test. Assays using BYDV GAV-infected wheat leaf crude extracts showed that the optimal dilutions of antibody 18A1 and the goat anti-mouse IgG/AP conjugate were 1:6,000 and 1:7,000, respectively. For detection of BYDV GAV in aphids, the optimal dilutions of antibody 18A1 and the goat anti-mouse IgG/HRP conjugate were 1:5,000 and 1:7,000, respectively. The specificity of the dot-ELISA was then confirmed using an extract from greenhouse BYDV GAV-infected wheat plants (a positive control) or extracts from the BYDV GPV-, BYDV PAV-, WYMV-, CWMV-, WDV- or BaYMV-infected or healthy wheat plants (negative controls) (Fig. 5a). Serial dilution assays showed that the dot-ELISA could be used to detect BYDV GAV in the infected wheat leaf extracts diluted at 1:5,120 (Fig. 5c). Similar results were also obtained by this assay using extracts from BYDV GAV viruliferous aphids (positive samples) or aphids fed on the BYDV GPV-, BYDV PAV-infected or healthy plants (negative controls, Fig. 5b).

### Serological assays for field sample

**Fig. 6:**
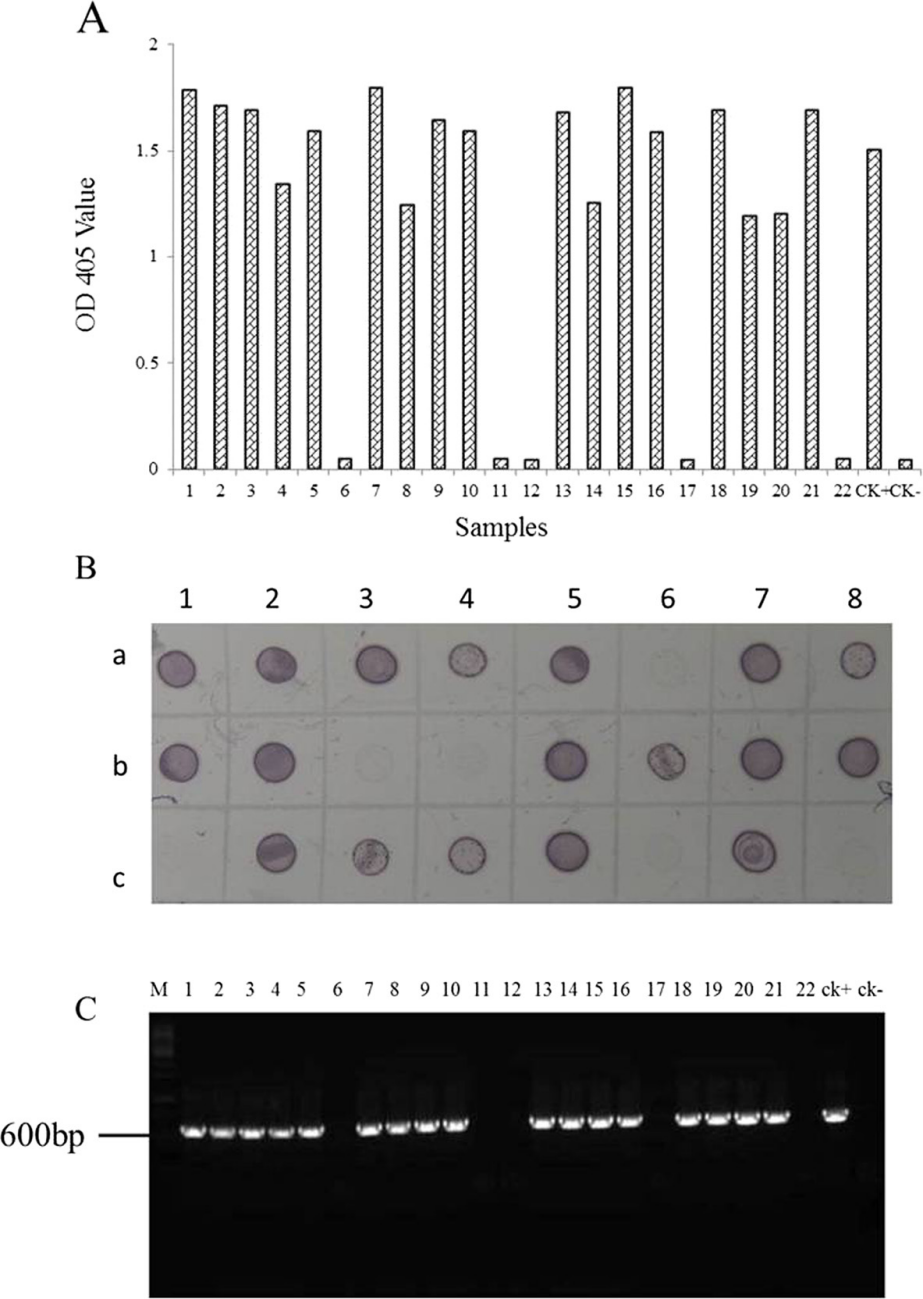
Detection of BYDV GAV in field samples by ACP-ELISA, dot -ELISA, and RT-PCR. **a**, Detection of BYDV GAV in field wheat samples through ACP-ELISA. Twenty two field wheat samples were loaded in wells on an ELISA plate. BYDV GAV-infected (CK+) and healthy wheat plants (CK-) were used as the positive and negative controls. **b**, Detection of BYDV GAV in wheat plant samples through dot-ELISA. Row A 1–8 were 1–8 samples shown in the panel A. B 1–8 were 9–16 samples shown in the panel A and C 1–6 were 17–22 samples shown in the panel A. Row C 7 and 8 were CK+ and CK- shown in the panel A. Dark purple color indicated a positive reaction. **c**, Detection of BYDV GAV in wheat samples through RT-PCR. Samples used in this assay were the same samples shown in the panel (**a**) and (**b**). BYDV GAV CP specific forward and reverse primers were used in this assay. Lane M, 1Kb DNA marker

**Fig. 7:**
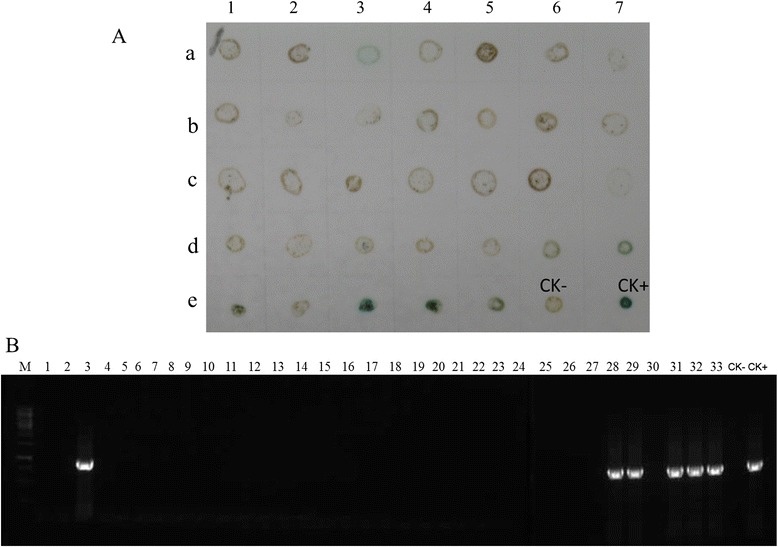
Detection of BYDV GAV in field aphids by dot-ELISA and RT-PCR. **a**, Detection of BYDV GAV in aphids through dot-ELISA. Row a1-7 to d1-7, and Row e 1–5 were 1–33 field aphids shown in the panel B. Row e 6 (CK-) and 7 (CK+) were from the non-viruliferous and viruliferous aphid, respectively, and used as controls. The membrane was probed with the monoclonal antibody 18A1 followed by the goat anti-mouse IgG/HRP conjugate. Blue color indicated a positive reaction. Light to dark brown color indicated a negative reaction. **b**, Detection of BYDV GAV in 33 field collected aphids by RT-PCR. Lane M, 1Kb DNA marker

To determine the usefulness of ACP-ELISA and dot-ELISA for field wheat and aphid samples, a total of 22 wheat samples and 33 aphids were collected from Hancheng in Shaanxi Province of China, and tested for BYDV GAV infection. Of the 22 wheat samples, 17 were tested positive for BYDV GAV infection by both ACP-ELISA and dot-ELISA (Fig. 6a, b). This result was validated through RT-PCR (Fig. 6c). Six of 33 aphid samples were tested positive for BYDV GAV infection by the dot-ELISA and later confirmed by RT-PCR (Fig. 7). The PCR products from these assays were cloned and sequenced. The sequencing results indicated that the BYDV strains found in these samples shared 94–99 % sequence identity with the known BYDV GAV strain *CP* sequences. This indicates that the GAV strain is the most common strain in Hancheng of Shaanxi Province.

## Discussion

In previous studies, BYDV GAV strain was characterized primarily through RT-PCR, reverse-transcription loop-mediated isothermal amplification assay (RT-LAMP) and dot-blot nucleic acid assays [16, 17]. Because these assays are time-consuming, high cost and requires specific instruments, we decided to develop simple and effective serological methods for BYDV GAV detection. It was reported previously that different BYDV strains could be distinguished using different monoclonal antibodies [5]. It was also reported that BYDV could be detected in oat leaf extracts and individual aphid vectors through an ELISA using a polyclonal antibody [18]. In a different study, the authors used Immunosorbent Electron Microscopy and three strain-specific monoclonal and two polyclonal antibodies to distinguish different BYDV strains [20]. In 1994, Makkouk and Comeau reported a tissue-blot immunoassay to detect BYDV infection in dried cereal tissues [21]. Several laboratories in China had attempted to produce polyclonal antibodies against BYDV. For example, Li et al. produced a polyclonal antibody against BYDV GPV movement protein. This antibody was, however, not reported for detection of BYDV in field samples [22]. In 2007, Xie et al. reported an antibody specific for the movement protein of BYDV GAV and used this antibody to detect BYDV GAV infection in wheat plant samples through Western blot [23]. Because monoclonal antibody is useful for detection of specific strains of plant viruses [24], we decided to produce monoclonal antibodies specific for BYDV GAV and to develop specific ACP-ELISA and dot-ELISA for its detection in field plant and aphid samples. We believe that ACP-ELISA and dot-ELISA presented in this paper can benefit researchers who are interested in BYDV epidemiology and/or wheat genotypes resistant to BYDV GAV infection.

In this study, three hybridoma lines secreting BYDV GAV specific monoclonal antibodies were generated. Using the prepared monoclonal antibody, sensitive ACP-ELISA and dot-ELISA were developed. Our results demonstrated that BYDV GAV could be detected by ACP-ELISA in 1:163,840 diluted wheat leaf extracts or by dot-ELISA in 1:5,120 diluted leaf extracts. Our result also showed that the dot-ELISA could be used to detect BYDV GAV in aphid vectors. Although the ACP-ELISA reported here was more sensitive for BYDV GAV detection than the dot-ELISA, we think that the dot-ELISA is particularly useful for field on-site detection of this virus due mainly to its simplicity and no requirement of expensive instruments.

Because the field samples tested in this study were all collected from the Shaanxi Province, the true distribution of BYDV GAV in China still remained to be determined. Production of monoclonal antibodies will be continued in order to establish serological assays for all the BYDV strains and for accurate forecast of BYDV in China. Accurate forecast of BYDV epidemiology is necessary for efficient BYDV management in field worldwide.

Martin et al. [14] reported that the molecular weight of the BYDV readthrough protein was about 60 kDa but not 72 kDa. They considered that this difference might be caused by anomalous running of the polypeptide in agarose gels due mainly to its protein conformation or to a posttranslational modification and/or degradation [14]. Our Western blot analyses using the three monoclonal antibodies indicated that the molecular weight of the detected protein was about 72 kDa (Fig. 3). Based on the molecular weight of this protein, we consider that these monoclonal antibodies are all specific for the readthrough protein but not the coat protein alone (22 kDa) or the protein encoded by the ORF5 (50 kDa).

## Conclusions

Both two developed serological assays in this study are suitable for sensitive, rapid and highthroughput detection of BYDV GAV in field wheat plants, and the dot-ELISA is suitable for the routine BYDV GAV detection of large-scale aphid vectors in BYDV GAV prevalent areas. The field survey demonstrated that BYDV GAV is widespread in Hancheng of Shaanxi Province.

## Materials and methods

### Sources of virus and field samples

Wheat plants showing virus-like symptoms were collected from wheat fields in Beijing in China, and a BYDV GAV strain was identified from these tissues through RT-PCR. The virus was inoculated to wheat plants via aphid transmission and the inoculated plants were maintained in a greenhouse till virus purification. To obtain viruliferous aphids, aphids were allowed to feed on the BYDV GAV-infected wheat plants maintained in a greenhouse for 5 days. These aphids used as positive controls during serological assays. BYDV GPV, BYDV PAV, WDV, WYMV, CWMV, BaYMV were originally collected from fields, identified by RT-PCR or PCR followed by nucleotide sequencing and maintained thereafter in wheat plants separately. Twenty two field wheat samples showing virus-like symptoms and thirty three aphids were collected from fields in Shaanxi Province of China in 2013, and stored at −80 °C till use.

### Preparation of monoclonal antibodies against BYDV GAV

BYDA GAV virion was purified from fresh BYDV GAV-infected wheat leaf tissues as described previously [25]. Purified BYDV GAV virion was loaded onto the formvar-coated grids and examined under an electron microscope (JEM −1200 EX, JEOL Ltd., Tokyo, Japan) prior to immunization of five six-week-old BALB/c mice purchased from the Shanghai Laboratory Animal Center, Chinese Academy of Sciences (Certificate of animal quality: Zhong Ke Dong Guan No.003) as described previously [26]. All animal experiments were carried out at the Research Center, the Laboratory of Animal Science, Zhejiang University of Traditional Chinese Medicine, Hangzhou, China. The experimental protocol was approved by the Animal Ethics Committee of Zhejiang University, Hangzhou, China.

Preparations of hybridomas secreting anti-BYDV GAV monoclonal antibodies and ascitic fluids were performed as described previously by Wu et al. [27]. Indirect- enzyme-linked immunosorbent assay (in-ELISA) was performed using purified BYDV virion as the coating antigen. This assay was used to determine the titer of monoclonal antibody in ascitic fluid. The isotypes of monoclonal antibodies were discriminated using a mouse monoclonal antibody isotyping kit as instructed by the manufacturer (Sigma-Aldrich, St. Louis, MO, USA). The specificity and sensitivity of the resulting antibodies were respectively confirmed by Western blot and ACP-ELISA as described previously [24, 26].

### Detection of BYDV GAV using ACP-ELISA

The optimal working concentration of anti-BYDV GAV monoclonal antibody and the goat anti-mouse IgG conjugated with alkaline phosphatase (goat anti-mouse IgG/AP, Sigma-Aldrich) for ACP-ELISA were determined by the phalanx test as described previously [26]. Detection of BYDV GAV in plant tissues was then performed following the procedure described by Wu et al. [24]. Briefly, 1 g wheat leaf tissues were ground in liquid nitrogen and then homogenized in 10 mL 0.05 mol · L^−1^ sodium bicarbonate buffer, pH 9.6. The extract was centrifuged for 3 min at 8000 × g and the resulting supernatant was two-fold diluted and loaded into wells (100 μL/well) of ELISA microplates followed by 2 h incubation at 37 °C or overnight at 4 °C. Wells contained crude extracts from the healthy (negative) or the BYDV GAV-infected (positive) wheat tissues were used as the controls. After 30 min blocking with a 0.01 mol · L^−1^ phosphate buffered saline (PBS, pH 7.4) containing 3 % dried skimmed milk, each well was incubated with a diluted monoclonal antibody for 1 h at 37 °C followed by an incubation for 1 h with the goat anti-mouse IgG/AP conjugate at 37 °C. The wells were washed 3–4 times with PBS containing 0.05 % tween-20 (PBST) between different steps. The detection signal was then visualized with p-nitrophenyl phosphate substrate as instructed (Sigma). The absorbance at OD_405_ was measured with a Microplate Reader Model 680 (BIO-RAD, Hercules, CA, USA). A sample was considered as positive when its absorbance value was at least three times greater than that for the negative controls.

### Dot-ELISA for BYDV GAV detection

Procedures of dot-ELISA were similar as that described previously [24]. Briefly, wheat crude extracts were prepared as described above. Individual aphid was placed on a Parafilm and then crashed in 5 μL PBS with the tip of a 0.5 mL eppendorf tube on the Parafilm. The wheat and mashed aphid extracts (2 μL each) were spotted onto nitrocellulose membranes (Amersham Biosciences, Bucks, UK,) and air-dried at RT. The negative and positive controls were extracts from the healthy and BYDV GAV-infected wheat plant tissues or from non-viruliferous and viruliferous aphids, respectively. The nitrocellulose membranes were soaked for 30 min in a PBST containing 5 % dried skimmed milk powder followed by 1 h incubation in the diluted monoclonal antibody and then 1 h incubation in the diluted goat anti-mouse IgG/AP or IgG/HRP solution. The membranes were washed 3–4 times with PBST between different steps. The detection signal was visualized by addition of NBT/BCIP (nitro–blue tetrazolium chloride/5-bromo-4-chloro-3-indolyl phosphate) or TMB (3, 3′, 5, 5′-tetramethylbenzidine) as instructed (Promega, Madison, WI, USA) for the AP and HRP conjugates. The positive signal visualized using NBT/BCIP was purple and the signal visualized using TMB is blue. Images of the membrane were taken after 10–20 min incubation in the substrate.

### RT-PCR and DNA sequencing

Total RNA was extracted from plant samples using the Trizol reagent as instructed by the manufacture and from aphids as described previously by Canning et al. [28]. Specific BYDV GAV forward primer (5′-ATGAATTCAGTAGGCCGTAGAA-3′, corresponding to the CP ORF nucleotide position 1–22) and reverse primer (5′-GTCTCGGTTTCCTCCAATGTG-3′, corresponding to the CP ORF nucleotide position 583–603) were designed according to the BYDV GAV CP ORF sequences available at the GenBank and used to detect the virus in leaf samples through RT-PCR as described [29]. The PCR products were cloned and sequenced individually. All the resulting sequences were aligned and analyzed using the Clustal W method provided by the DNASTAR software (Version 7.0, DNAStar Inc., Madison, WI, USA).

## References

[1] Lister RM, Ranieri R (1995). Distribution and economic importance of barley yellow dwarf. See Ref.

[2] Miller WA, Rasochova L (1997). Barley yellow dwarf viruses. Annu Rev Phytopathol.

[3] Oswald JW, Houston BR (1951). A new virus disease of cereals, transmitted by aphid. Plant Dis Rep.

[4] Shah SJA, Bashir M, Manzoor N (2012). A Review on Barley Yellow Dwarf Virus. Crop Production for Agricultural Improvement.

[5] Rochow WF (1979). Comparative diagnosis of BYDV by serological and aphid transmission tests. Plant Dis Rep.

[6] Rochow WF (1969). Biological properties of four isolates of BYDV. Phytopathology.

[7] Zhou GH, Zhang SX, Qian YT (1987). Identification and application of four strains of wheat yellow dwarf virus. Sci Agric Sin.

[8] Zhang SX, Zhou GH (1987). Identification on strain of wheat yellow dwarf virus (WYDV) transmitted by *Schizaphis graminum* and *Rhopalosiphum padi*. Acta Phytopathologica Sinica.

[9] Wiese MV. 1977. Compendium of wheat diseases. American Phytopathological Society. St. Paul, Minnesota, USA

[10] Burnett PA (1990). World perspectives on barley yellow dwarf virus.

[11] Khetarpal PK, Kumar J, Beuve M, Parakh DB, Nath R (1994). Outbreak of MAV-type barley yellow dwarf virus on wheat in the Garwal Hills in India. Plant Pathol.

[12] Gill CC, Chong J (1979). Cytopathological evidence for the division of barley yellow dwarf virus isolates into two subgroups. Virology.

[13] Miller WA, Waterhouse PM, Gerlach WL (1988). Sequence and organization of barley yellow dwarf virus genomic RNA. Nucleic Acids Res.

[14] Martin RR, Keese PK, Young MJ, Waterhouse PM, Gerlach WL (1990). Evolution and molecular biology of luteoviruses. Annu Rev Phytopathol.

[15] Ueng PP, Vincent JR, Kawata EE (1992). Nucleotide sequence analysis of genomes of the MAV-PS1 and P-PAV isolates of barley yellow dwarf virus. J Gen Virol.

[16] Svanella-Dumas L, Candresse T, Hulle M, Marais A (2013). Distribution of Barley yellow dwarf virus-PAV in the sub-Antarctic Kerguelen Islands and characterization of two new *Luteovirus* species. Plos one.

[17] Liu Y, Sun B, Wang X, Zheng C, Zhou G (2007). Three digoxigenin-labeled cDNA probes for specific detection of the natural population of Barley yellow dwarf viruses in China by dot-blot hybridization. J Virol Methods.

[18] Torrance L (1987). Use of enzyme amplification in an ELISA to increase sensitivity of detection of barley yellow dwarf virus in oats and in individual vector aphids. J Virol Methods.

[19] Hall GS, Peters JS, Little DP, Power AG (2010). Plant community diversity influences vector behavior and Barley yellow dwarf virus population structure. Plant Pathol.

[20] Forde SMD (1989). Strain differentiation of barley yellow dwarf virus isolates using specific monoclonal antibodies in immunosorbent electron microscopy. J Virol Methods.

[21] Makkouk KM, Comeau A (1994). Evaluation of various methods for the detection of barley yellow dwarf virus by the tissue-blot immunoassay and its use for virus detection in cereals inoculated at different growth stages. Eur J Plant Pathol.

[22] Li YL, Wu MS, Wang ZY, Zhang WW, Cheng ZM (2004). The ORF4 gene of barley yellow dwarf virus GPV expressed in *e. coli* and the specific antiserum prepared. Acta Phytopathologica Sinica.

[23] Xie JJ, Wang XF, Liu Y, Peng YF, Zhou GH (2007). The movement protein of BYDV GAV expressed, purificated and antiserum prepared, tested. Chinese Soc Plant Pathol.

[24] Wu JX, Ni YQ, Liu H, Ding M, Zhou XP (2014). Monoclonal antibody-based serological assays and immunocapture-RT-PCR for detecting Rice dwarf virus in field rice plants and leafhopper vectors. J Virol Methods.

[25] Rochow WF, Brakke MK (1964). Purification of barley yellow dwarf virus. Virology.

[26] Shang HL, Xie Y, Zhou XP, Qian YJ, Wu JX (2011). Monoclonal antibody-based serological methods for detection of Cucumber green mottle mosaic virus. Virol J.

[27] Wu JX, Yu C, Yang CY, Deng FL, Zhou XP (2009). Monoclonal antibodies against the recombinant nucleocapsid protein of tomato spotted wilt virus and its application in the virus detection. J Phytopathol.

[28] Canning ESG, Penrose MJ, Barker I, Coates D (1996). Improved detection of barley yellow dwarf virus in single aphids using RT-PCR. J Virol Methods.

[29] Wang X, Chang S, Jin Z, Li L, Zhou G (2000). Nucleotide sequences of the coat protein and readthrough protein genes of the Chinese GAV isolate of barley yellow dwarf virus. Acta Virol.

